# Short-Term Bone Healing in Anterior Maxillary Sockets Using L-PRF With or Without Synthetic HA/β-TCP: A Randomized Clinical Trial

**DOI:** 10.3390/jfb17010006

**Published:** 2025-12-22

**Authors:** Pricila da Silva Gusmão, Cássia Pereira da Silva, Víctor Ravelo, Akinori Cardozo Nagato, Sergio Olate, Henrique Duque

**Affiliations:** 1 Faculty of Odontology, Federal University of Juiz de Fora, Juiz de Fora 36036-900, MG, Brazil; pricilasgusmao@gmail.com (P.d.S.G.); cassiodontoujfj@gmail.com (C.P.d.S.); akinori.nagato@ufjf.br (A.C.N.); henrique.duque@ufjf.br (H.D.); 2 PhD Program, Center for Research in Morphology and Surgery (CEMyQ), Universidad de La Frontera, Temuco 4780000, Chile; victor.ravelo.s@gmail.com; 3 Facultad de Ciencias de la Salud, Universidad Autónoma de Chile, Temuco 4810101, Chile; 4 Department of Oral Diagnosis, Division of Oral and Maxillofacial Surgery, State University of Campinas, Piracicaba 13414-903, SP, Brazil

**Keywords:** alveolar ridge preservation, leukocyte- and platelet-rich fibrin (L-PRF), hydroxyapatite with β-tricalcium phosphate (HA/β-TCP), bone regeneration

## Abstract

Tooth extraction induces changes in both hard and soft tissues, which may compromise implant placement. Leukocyte- and platelet-rich fibrin (L-PRF) is used to promote tissue healing, either alone or in combination with other grafting materials. Objective: This study aimed to compare post-extraction socket healing using L-PRF alone or combined with a biphasic calcium phosphate graft (HA/β-TCP) after eight weeks. Materials and Methods: 15 patients, both sexes, mean age 56.7 ± 8.2 years, requiring alveolar ridge preservation after single-rooted tooth extraction for subsequent implant placement, were included. Sockets were randomly assigned to four groups: control with blood clot only (CTR), autogenous bone graft (AB), L-PRF membrane (LPRF), and L-PRF combined with HA/β-TCP (LPRFHA). The protocol consisted of tooth extraction and immediate graft placement, followed by bone biopsy at 8 weeks for histomorphometric analysis and implant installation. New Bone Formation (NBF) was quantified from ten photomicrographs per sample using ImageJ software (version 1.54, 5 February 2025). One-way ANOVA with Bonferroni post hoc tests was applied, with statistical significance set at *p* ≤ 0.05. Results: A significant difference in NBF (%) was observed between the control and LPRFHA groups (*p* = 0.014), with greater bone formation in the control group (62.4 ± 18.6%) compared with LPRFHA (55.8 ± 17.2%; *p* = 0.012). No significant differences were found among AB, LPRF, and LPRFHA groups. LPRF and AB showed comparable bone formation (60.2 ± 17.5% and 60.1 ± 20.0%, respectively). Conclusions: L-PRF, either alone or combined with HA/β-TCP, can be used for alveolar ridge preservation in maxillary sockets. L-PRF, alone or with synthetic HA/β-TCP graft, effectively preserves the anterior maxillary ridge for early loading at eight weeks. All treatments achieved bone formation for implant placement, with the blood clot alone showing superior results.

## 1. Introduction

Tooth extraction initiates a complex metabolic process regulated by growth factors, which promotes new bone formation through osteogenesis, while simultaneously inducing resorption of the preexisting tissue and its replacement by mature bone during remodeling [1]. In the case of alveolar ridge remodeling, a reduction of 3–5 mm in horizontal dimensions and 1–3 mm in vertical dimensions occurs within the first three to six months, which may compromise implant rehabilitation [2]. To address this challenge, several procedures have been proposed to optimize bone regeneration, including socket grafting techniques using autologous, allogeneic, xenogeneic, or alloplastic bone substitutes, as well as biologically active materials such as growth factors [3,4].

Autogenous bone is considered the gold standard for bone grafting due to its osteogenic, osteoinductive, and osteoconductive properties. However, alternatives are necessary because of surgical morbidity and the limited amount of graft material available for reconstruction [5]. Current alternatives such as allografts, xenografts, and synthetic biomaterials act as structural scaffolds that facilitate osteoconduction [6]. Nevertheless, both allografts and xenografts may trigger immune responses that modulate bone regeneration, in which acute inflammation plays a critical role in resorption and the reduction of bone volume [7]. In contrast, alloplastic grafts do not induce inflammatory responses, which provides volumetric stability but limits degradation and bone remodeling within their internal surface, ultimately leading to reduced biomechanical response and an increased risk of fracture [8].

**Figure 1:**
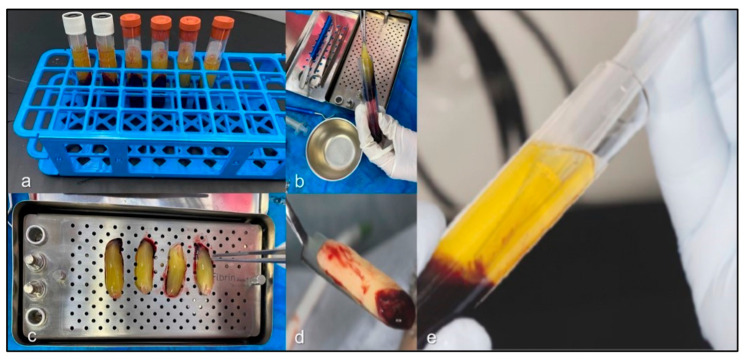
Stages of particularly leukocyte- and platelet-rich fibrin (L-PRF) processing: (**a**) L-PRF matrix; (**b**) fibrin clot extraction after centrifugation; (**c**) drainage of excess exudate in the L-PRF box; (**d**) membrane ready for surgical use; (**e**) fraction fluid liquid (FFL) used as a polymerizing agent for LPRFHA preparation.

Over the past decades, research in biomaterial-based tissue engineering has gained increasing relevance in both medical and dental fields. The application of platelet-enriched materials has shown positive outcomes, particularly leukocyte- and platelet-rich fibrin (L-PRF), which stands out as an osteoinductive alternative through cytokines and growth factors with the potential to stimulate tissue regeneration [9,10,11,12,13] (Figure 1).

Although L-PRF is not considered an osteoinductive material on its own, its combination with bone grafts and other biomaterials has shown favorable outcomes in enhancing bone regeneration and soft tissue healing, acting as a biological connector that promotes cell recruitment, osteoprogenitor migration, and neoangiogenesis [14,15].

**Figure 2:**
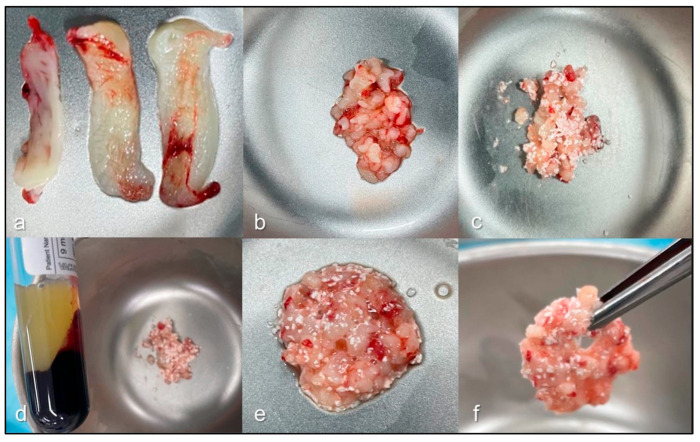
Preparation process of leukocyte- and platelet-rich fibrin and hydroxyapatite (LPRFHA). Initially, leukocyte- and platelet-rich fibrin (L-PRF) membranes are obtained after the dehydration process (**a**), ideally providing four units according to the established protocol. One of these membranes is finely chopped (**b**) and mixed with the bioceramic biomaterial (**c**). Next, fraction fluid liquid (FFL) is added to this mixture (**d**), initiating the agglutination process (**e**). Finally, a cohesive and homogeneous material, known as sticky bone (**f**), is obtained, ready for use in bone regeneration procedures.

The objective of this study was to compare the healing response of L-PRF alone and in combination with the synthetic graft HA/β-TCP (Figure 2) in bone regeneration of human maxillary sockets after an eight-week post-extraction period.

## 2. Materials and Methods

**Figure 3:**
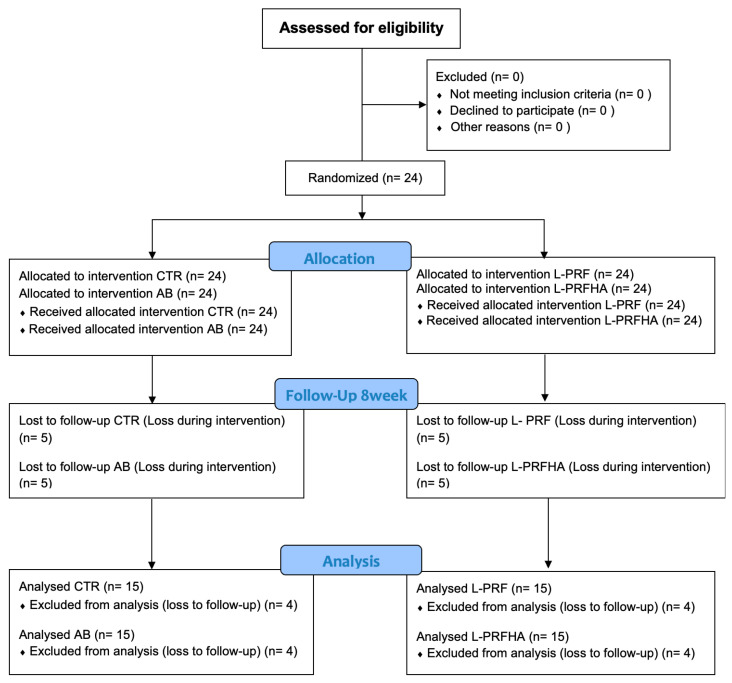
CONSORT flow diagram.

A controlled, prospective, double-blind clinical trial registration number U1111-1312-1819 (Brazilian Registration of Clinical Trials—ReBEC) (Figure 3), was conducted to evaluate the efficacy of L-PRF, used alone or in combination with a synthetic HA/β-TCP graft, in the bone healing of fresh human maxillary sockets after eight weeks. The protocol was approved by the Ethics Committee of the Federal University of Juiz de Fora (Approval No. 4,875,915, 30 July 2021). The alveoli were randomized using a simple random sequence into four groups using Microsoft Excel^®^ (Microsoft Office Home 2019). The random allocation was concealed from the participants and the involved professionals, including the surgeon responsible for biopsy collection and the investigator in charge of slide reading. The randomization sequence was revealed only to the surgeon, who performed the extractions and managed the alveoli (control and experimental groups). After image acquisition and processing, the randomization was revealed to the investigators to assign the data to the corresponding groups and subsequently quantify the New Bone Formation (BRA).

Patients classified as ASA I or II requiring extraction of at least four single-rooted teeth in the maxillary region were included. All participants presented a bone classification suitable for type 2 implant placement [16], with sufficient height and width to achieve primary stability. The Extraction Defect Sounding (EDS) classification assesses the extent of alveolar bone loss following tooth extraction, helping to determine the necessity of regenerative interventions. In this study, the sockets showed moderate bone defects, typically involving partial loss of one alveolar wall, and required only minimal bone regeneration procedures for implant placement (EDS-2/EDS-3) [17].

Exclusion criteria included uncontrolled systemic diseases, smoking >10 cigarettes/day, use of drugs affecting bone metabolism, hematologic disorders, local infections, or the need for extraction of multi-rooted teeth. Extractions were performed using an atraumatic, flapless technique, and sockets were randomly assigned to four groups: (a) control without graft (CTR), (b) particulate autogenous bone (AB), (c) L-PRF alone (LPRF), and (d) sticky bone (LPRFHA) a mixture of L-PRF and HA/β-TCP (150–425 µm, Osteosynt^®^ (Teknimed, Toulouse, France). Randomization was kept blinded for both participants and evaluators. L-PRF was obtained from peripheral blood collected in additive-free tubes and centrifuged at 2700 rpm for 12 min (750 g).

The clots were processed into standardized membranes, and the fraction fluid liquid (FFL) was used to mix with the biomaterial in the sticky bone group. The ratio was one membrane per 0.5 g of biomaterial, resulting in a cohesive graft. Following socket grafting, sutures were placed using polyglactin 910. Patients received standard postoperative medication, including analgesics, antibiotics, anti-inflammatories, and 0.12% chlorhexidine for six weeks.

After satisfactory healing without relevant complications, the final sample comprised 15 patients and 60 sockets. At eight weeks, a customized guide was fabricated for bone biopsy collection and subsequent implant placement. Samples were fixed in 10% formalin and processed for light microscopy using routine preparation (10% EDTA decalcification, paraffin embedding, 3 µm sections, and H&E staining). NBF was measured in μm^2^ using ImageJ (National Institutes of Health, Bethesda, MD, USA), analyzing only particles within a size range defined by the software’s “Size” command.

A pilot study involving 24 alveoli was conducted to estimate the effect size, which was determined to be 0.376. Based on this value and using a significance level of 5% and a statistical power of 80%, the sample size calculation was performed using the G*Power 3.1.9.4 software (Heinrich-Heine-Universität, Düsseldorf, Germany), considering a repeated-measures analysis of variance (ANOVA) model. The calculation indicated the need to include at least 12 participants (48 alveoli). To compensate for a potential loss to follow-up estimated at 20%, a minimum final sample of 15 participants (60 alveoli) was determined. Statistical analysis included one-way ANOVA with Bonferroni post hoc tests, or, when normality assumptions were not met, Kruskal-Wallis with Dunn or Sidak post hoc tests. Statistical significance was set at *p* < 0.05 (95% CI). Data were processed in STATA 15 (StataCorp LLC, College Station, TX, USA), and graphs were generated using STATISTICA 12.0 (StatSoft Inc., Tulsa, OK, USA).

## 3. Results

Fifteen subjects (7 women and 8 men), with a mean age of 56.7 ± 8.2 years, were analyzed, and sockets were randomly assigned to four treatment groups: CTR, AB, L-PRF, and LPRFHA. After eight weeks, histomorphometric analyses were performed on the biopsy samples, evaluating the New Bone Formation (BRA) in μm^2^ and as a percentage. The data showed a normal distribution, allowing valid statistical comparisons between groups, which presented a homogeneous distribution of BRA percentages: AB (25.0%), CTR (25.2%), LPRF (24.9%), and LPRFHA (25.0%).

**Figure 4:**
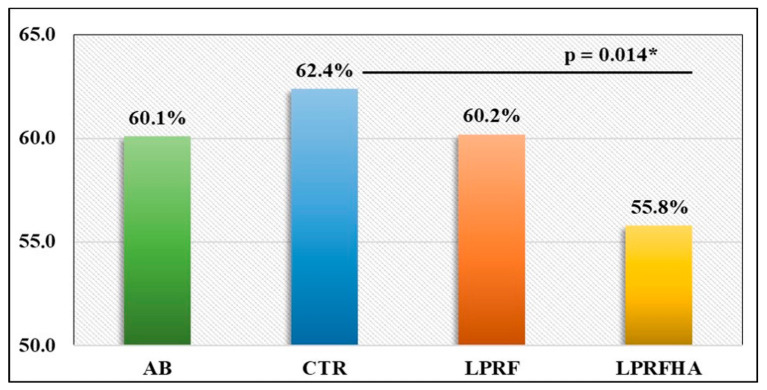
Comparison of mean NBF (New Bone Formation) (%) rates between groups: AB (autogenous bone); CTR (control); LPRF (L-PRF membrane); LPRFHA (L-PRF combined with synthetic HA/β-TCP graft); * (significant difference).

Statistical analysis using one-way ANOVA revealed a significant difference between the CTR and the LPRFHA group (*p* = 0.014), which was confirmed by Bonferroni post hoc testing. The CTR group exhibited the highest mean NBF percentage (62.4 ± 18.6%), compared with the lowest in the LPRFHA group (55.8 ± 17.2%) (*p* = 0.012). No significant differences were observed between the groups receiving L-PRF alone or in combination with the synthetic graft (Table 1, Figure 4).

**Figure 5:**
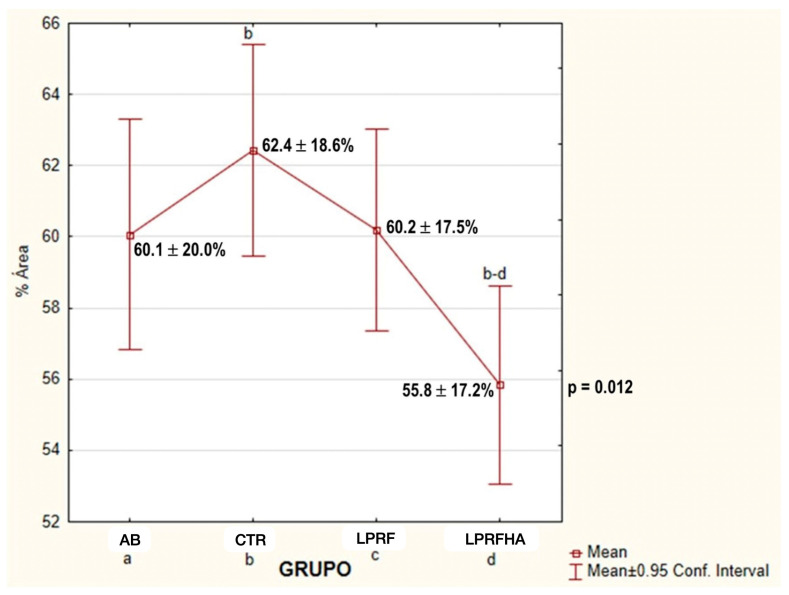
Multiple comparison of mean NBF (%) between groups: AB (autogenous bone); CTR (control); LPRF (L-PRF membrane); LPRFHA (L-PRF combined with synthetic HA/β-TCP graft). a: AB (autogenous bone); b: CTR (control); c: LPRF (L-PRF membrane); d: LPRFHA (L-PRF combined with synthetic HA/β-TCP graft).

Furthermore, the LPRF and AB groups showed similar NBF results, with values of 60.2% and 60.1%, respectively. These similarities are reflected in the multiple comparison analyses (Figure 5), where significant differences between groups are detailed (*p* = 0.012).

Regarding the descriptive analysis of the histological samples, notable morphological differences were observed in the organization of newly formed bone and tissue integration. In the CTR, AB, and L-PRF groups, mature lamellar bone was present, with densely eosinophilic trabeculae containing osteocytes within well-defined lacunae (indicated by yellow arrows), bordered by loose, vascularized fibrous connective tissue. In the AB group, autogenous bone exhibited an organized architecture, with well-formed Haversian systems and osteonal structures, indicative of active remodeling. In the L-PRF group, the interface between newly formed bone and soft tissue was continuous, with no signs of inflammation or residual particulate material, demonstrating favorable tissue integration.

**Figure 6:**
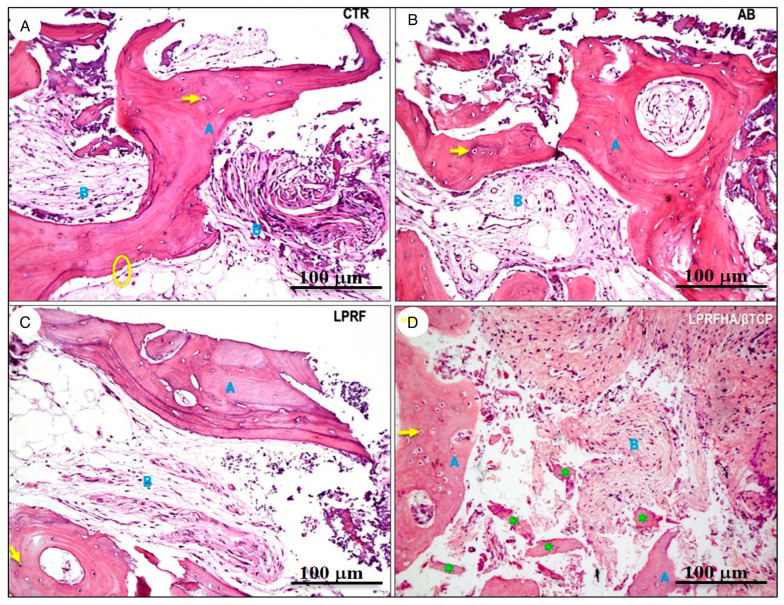
Histological sections stained with hematoxylin and eosin (H&E, 10× magnification) show different experimental conditions: (**A**) control (CTR), (**B**) autogenous bone (AB), (**C**) L-PRF membrane (L-PRF), and (**D**) L-PRF combined with synthetic HA/β-TCP graft. The bone matrix (blue A) is clearly identified alongside connective tissue (blue B). Osteocytes are indicated with arrows (→); osteoblasts with circles (○); and residual bone graft particles with asterisks (*).

In the LPRFHA/β-TCP group, the bone tissue exhibited an immature and fragmented arrangement, with lower trabecular density and areas of forming osteoid matrix. Multiple basophilic spherical particles corresponding to remnants of the synthetic HA/β-TCP biomaterial (green asterisks) were observed, partially integrated into the perivascular connective tissue (Figure 6).

## 4. Discussion

This study allowed comparison of the materials’ influence on early bone development and their potential suitability for implant osseointegration. Synthetic bone substitutes are commercially available in various forms, exhibiting differences in physicochemical properties, including porosity, particle or block size and morphology, as well as degradation rates. Despite these variations, their capacity is limited to osteoconduction, lacking osteoinductive potential [18,19]. HA/β-TCP-based substitutes act as biocompatible, osteoconductive scaffolds that facilitate bone repair, with the aim of being progressively degraded and replaced by new bone at the defect site [20,21,22].

Although the growth factors present in L-PRF are gradually released over a period of up to 14 days, supported by a fibrillar matrix that acts as a natural biological scaffold, this fibrin matrix also restricts fibrous connective tissue infiltration and promotes cell migration and differentiation key processes for regeneration [23]. Several studies [23,24,25] report that L-PRF, when combined with bone biomaterials, functions as a biological connector that enhances cell adhesion, growth factor release, and vascularization at the graft site, thereby potentiating osteoinductive properties and accelerating new bone formation. The primary aim of this study was to evaluate the presence of bone and specific characteristics of the bone structure that would be in contact with the implant. After the 8-week healing period, bone formation was assessed in sockets treated as control, hydroxyapatite, or L-PRF, alone or in combination with HA/β-TCP. Results showed lower bone formation in the L-PRF + HA/β-TCP group, suggesting that the addition of an alloplastic material may have early reduction of osteogenesis, while no significant differences when compared to the use of L-PRF or autologous blood clot (AB).

In an experimental rabbit calvarial defect model, Acar et al. [23] reported that the group treated with the HA/β-TCP + L-PRF combination exhibited greater new bone formation at eight weeks, likely attributable to the sustained release of growth factors such as PDGF, TGF-β, and VEGF from L-PRF. Similarly, Nacopoulos et al. [14] evaluated bone regeneration in an animal model using defects treated with L-PRF, HA/β-TCP, and their combination. At three months, the combined group showed significantly higher bone density, demonstrating a synergistic effect that enhances angiogenesis and stimulates bone formation and remodeling. Baghele et al. [26] conducted a clinical study in patients undergoing tooth extractions, comparing bone regeneration using L-PRF alone and in combination with hydroxyapatite/β-tricalcium phosphate (HA/β-TCP). At six months, both groups showed significant improvements in bone volume and density, with no statistically relevant differences between them, suggesting that the addition of the biomaterial did not provide any further significant benefits.

In the present study, similar values were observed, with the groups treated with L-PRF alone (60.2%) and L-PRF + HA/β-TCP (55.8%) showing no statistically significant differences in bone formation. The lower proportion observed in the combined group could be explained by space occupation by the biomaterial, which requires a vascularization process for integration, as well as by the slow degradation of hydroxyapatite, thus limiting the osteogenic potential of L-PRF. Other factors, such as differences in biological study models, individual characteristics, alveolar defect morphology and microstructure, as well as variations in the bone substitutes used in other studies, may account for the observed differences.

Previous studies [27,28] have shown that the use of alveolar ridge preservation after tooth extraction whether using the clot, L-PRF, or growth factors without bone grafts or biomaterials does not result in significant differences in primary implant stability. However, during the secondary stability phase, the L-PRF-treated group exhibited higher ISQ values, suggesting that changes in bone response to implant loading may occur in the medium term.

A randomized clinical trial, Elsheikh et al. [29] independently compared the use of L-PRF, xenograft, and alloplastic material, followed by implant placement in premolar sockets. Results showed that during implant placement and at 6- and 18-month evaluations, there were no significant differences between groups; however, ISQ values were higher in the xenograft and alloplastic material groups compared with the L-PRF-only group. Similarly, Shahbaz-Alam et al. [30] reported comparable findings when comparing alloplastic biomaterial, L-PRF, and their combination, observing similar primary stability across the three groups. It is noteworthy that in both clinical trials, participants had residual bone heights between 2 and 5 mm and insertion torque values ranging from 35 to 40 Ncm. These findings suggest that the amount of residual bone is a determining factor for primary implant stability, whereas the use of bone grafts acts as a complementary measure that does not affect this initial phase but may contribute to enhanced secondary stability.

L-PRF promotes soft tissue healing and regeneration through the release of growth factors, improving keratinized mucosa thickness and gingival stability associated with favorable aesthetic outcomes in color and texture for root coverage or peri-implant areas [31]. Additionally, it reduces inflammation, postoperative pain, and morbidity by avoiding a donor site [32]. Hajibagheri et al. [33], through a meta-analysis, demonstrated that PRF enables faster and higher-quality soft tissue repair during the first two weeks post-intervention, resulting in improved aesthetic outcomes. Regarding hard tissues, L-PRF did not achieve vertical alveolar preservation but did help maintain bone width and prevent postoperative complications within the first three months [33]. These results are not directly comparable to those of the present study, as aesthetic or soft tissue parameters were not included; however, the authors agree that L-PRF contributes to the enhancement of peripheral soft tissues and that its use in alveolar ridge preservation may also support the maintenance and stability of peri-implant soft tissue.

L-PRF is an autologous material with regenerative properties, without the costs associated with other materials or membranes, eliminating the need for autologous donor sites [34,35]. A notable feature of L-PRF is its high leukocyte concentration, which plays a key role in pathogen inhibition [3,9]. In our study, no participants experienced postoperative clinical complications (pain, swelling, or infection), regardless of the treatment received. The highest percentage of bone repair was observed in the control group (CTR = 62.4%), followed by L-PRF (60.2%) and autogenous bone (AB, 60.1%), with no statistically significant differences between them. These findings are consistent with previous reports [23], which highlighted that, in addition to its hemostatic function, platelets release factors such as PDGF, VEGF, and TGF-β, stimulating the expression of osteogenic markers in osteoblasts. Given its bone repair capacity comparable to autogenous grafts, L-PRF can be considered a viable alternative.

In this study, the use of sticky bone demonstrated a significantly lower percentage of bone formation (55.8 ± 17.2%) compared with the control sockets (CTR: 62.4 ± 18.6%). Maintaining the blood clot alone in post-extraction sockets could be more efficient for new bone formation than using biomaterials, as it eliminates the need for degradation or remodeling and the formation is exclusively bone. Considering that in the present study a trephine was used to harvest bone from the implant surgical site, it is not possible to guarantee that using only the clot would maintain the alveolar volume, since only the trephine contents were analyzed. The presence of biomaterials within the socket may help preserve ridge volume without significantly limiting new bone formation when used in combination with L-PRF. Although the evaluated healing period is relatively short to fully assess bone consolidation, this approach remains a viable alternative to allow early loading while promoting stability in the vestibular bone plate and facilitating implant placement.

It was confirmed that after eight weeks, the particles of this material, like those of the autogenous graft, maintained their structure and did not impede early bone formation. In this context, the allograft and xenograft exhibit a shorter degradation time compared to alloplastic biomaterials, with β-TCP remaining in an active degradation phase for approximately 12 months [36]. In contrast, HA displays a longer degradation time than β-TCP, suggesting that HA/β-TCP incorporation may necessitate an extended period to achieve complete bone remodeling [37], although this process is also influenced by the material’s microstructure.

Eight weeks is a short period to expect complete bone repair and material degradation, but it is sufficient to identify new soft tissue formation to cover the socket and allow implant surgery with predictable aesthetic outcomes. The presence of biomaterial within the socket does not interfere with osseointegration and may help maintain the soft tissue support necessary for the future function of the implant in the anterior region. Thus, the use of biomaterials under these conditions contributes to preserving ridge volume and stabilizing the soft tissues that support esthetics [16]. Additionally, a six-month randomized controlled clinical trial [26] evaluated the use of an alloplastic graft (HA/β-TCP), applied either alone or in combination with L-PRF, in intraosseous defects of patients with periodontitis. The results showed that the combination of HA/β-TCP with L-PRF produced more favorable outcomes compared to the graft used alone, with improvements observed in radiographic bone fill parameters. Although these studies have different objectives, it is important to emphasize that bone remodeling is closely associated with the duration of the follow-up period. On the other hand, the study by Wei et al. [38] evaluated the clinical efficacy and safety of the bone substitute composed of ErhBMP-2/BioCaP/β-TCP through a proof-of-concept randomized clinical trial applied to a post-extraction socket-healing model. This investigation was conducted in a single premolar site per patient under controlled conditions, including vestibular and palatal bone thicknesses greater than in the anterior area, thereby providing consistent evidence regarding the performance of the biomaterial in a defined clinical setting. In contrast, the present study was carried out in four sites per individual, all located in the anterior region. This approach offers a broader and complementary methodological scope, allowing for a more comprehensive characterization of the tissue response in anatomical and functional scenarios.

Volumetric analysis of socket preservation using CBCT was not performed, as the two-month follow-up period would likely not provide a clear assessment of bone formation in this research.

L-PRF, both alone and in combination with the synthetic HA/β-TCP graft, can be used for alveolar ridge preservation in maxillary sockets. However, alveolar anatomy is a key determinant in bone healing due to variations in width, height, thickness, and density of the bone walls, as well as the possible presence of fenestrations or dehiscences, which complicates precise quantification of the graft’s effect on regeneration. In addition, graft location influences the pattern of bone formation, which begins at the lateral wall of the socket and progresses toward the center [39]. Despite the short follow-up period inherent to the early loading protocol, there is a recognized need to include assessments of vertical bone height using cone beam computed tomography to quantitatively determine the amount of vertical bone preserved, as well as to evaluate implant insertion torque and the Implant Stability Quotient in relation to different biomaterials.

This randomized clinical trial has certain limitations that should be considered when interpreting the results. The small sample size may have limited the statistical power and increased imprecision, particularly given that the effect size was not calculated for each group. Although proper randomization was applied, incomplete operator blinding and individual biological variability could have influenced the outcomes. An adjustment for multiplicity of comparisons was performed to reduce the risk of type I errors; however, future studies should incorporate effect size estimation for each outcome, with larger sample sizes and longer follow-up periods to strengthen the evidence.

Another limitation of this study was the absence of cellular and platelet counts via complete blood count to assess the cellular profile, as blood composition can influence tissue regeneration, including platelets, leukocytes, and erythrocytes. In this context, platelet count could be related to L-PRF membrane size, whereas higher peripheral erythrocyte counts may be associated with smaller L-PRF membranes [40]. Additionally, qualitative and quantitative analyses using specific histological collagen stains could reveal differences in tissue organization between experimental groups, and immunostaining would be useful to identify cell types characteristic of inflammatory granulation and to distinguish intrinsic tissue cellularity.

Finally, it is essential to advance controlled and randomized clinical trials with standardized protocols in humans to confirm whether the findings of this research support the use of L-PRF as a standalone biomaterial in fresh maxillary sockets, promoting bone repair within an eight-week period.

## 5. Conclusions

In conclusion, early implantation at eight weeks can be successfully achieved using L-PRF, either alone or in combination with a synthetic HA/β-TCP graft, for alveolar ridge preservation in the anterior maxilla. All treatments resulted in over 50% new bone formation, ensuring adequate bone volume for subsequent implant placement, with the blood clot alone demonstrating superior bone formation compared to the graft combination. Future research should focus on long-term implant stability and prosthetic outcomes.

## Figures and Tables

**Table 1:** Mean (%) values and measures of dispersion for New Bone Formation (NBF) across groups.

Grupos	NBF (%)			*p*-Value
	Mean ± SD	Median	Minimum–Maximum	
AB	60.1 ± 20.0	58.4	15.6–99.2	0.014 *
CTR	62.4 ± 18.6 *	64.2	21.6–97.6	
LPRF	60.2 ± 17.5	63.8	17.2–92.9	
LPRFHA	55.8 ± 17.2 *	54.9	12.7–96.1	

**Abbreviations:** NBF (New Bone Formation); % (percentage); AB (autogenous bone); CTR (control); LPRF (L-PRF membrane); LPRFHA (L-PRF combined with synthetic HA/β-TCP graft); SD (standard deviation); * (significant difference).

## Data Availability

The data are available upon request from the corresponding author.
